# Socio-economic differentials in minimum dietary diversity among young children in South-East Asia: evidence from Demographic and Health Surveys

**DOI:** 10.1017/S1368980018002173

**Published:** 2018-09-04

**Authors:** Chloe M Harvey, Marie-Louise Newell, Sabu S Padmadas

**Affiliations:** 1 Department of Social Statistics and Demography, University of Southampton, Southampton SO17 1BJ, UK; 2 Global Health Research Institute, Human Development and Health, Faculty of Medicine, University of Southampton, Southampton, UK

**Keywords:** Young children, Minimum dietary diversity, Socio-economic, Demographic and Health Surveys, South-East Asia

## Abstract

**Objective:**

To investigate the socio-economic differentials underlying minimum dietary diversity (MDD) among children aged 6–23 months in three economically diverse South-East Asian countries.

**Design:**

The outcome variable MDD was defined as the proportion of children aged 6–23 months who received foods from four of the seven recommended food groups within the 24 h prior to interview. The association between socio-economic factors and MDD, adjusting for relevant characteristics, was examined using logistic regression.

**Setting:**

We used cross-sectional population data from recent Demographic and Health Surveys from Cambodia (2014), Myanmar (2015–16) and Indonesia (2012).

**Subjects:**

Total of 8364 children aged 6–23 months.

**Results:**

Approximately half of all children met the MDD, varying from 47·7 % in Cambodia (*n* 1023) to 58·2 % in Indonesia (*n* 2907) and 24·6 % in Myanmar (*n* 301). The likelihood (adjusted OR; 95 % CI) of meeting MDD increased for children in the richest households (Cambodia: 2·4; 1·7, 3·4; Myanmar: 1·8; 1·1, 3·0; Indonesia: 2·0; 1·6, 2·5) and those residing in urban areas (Cambodia: 1·4; 1·1, 1·9; Myanmar: 1·7; 1·2, 2·4; Indonesia: 1·7; 1·5, 1·9). MDD deprivation was most severe among children from the poorest households in rural areas. The association between mother’s labour force participation and MDD was positive in all three countries but reached significance only in Indonesia (1·3; 1·1, 1·5).

**Conclusions:**

MDD deprivation among young children was significantly high in socio-economically disadvantaged families in all three study settings. MDD requirements are not being met for approximately half of young children in these three South-East Asian countries.

Optimal nutrition during early life improves child survival^(^ ^1^ ^)^ and reduces risk of chronic, lifestyle-related diseases^(^ ^2^ ^–^ ^6^ ^)^. Despite strong economic growth^(^ ^7^ ^)^ and steady reductions in mortality among children <5 years of age (under-5s) in South-East Asia^(^ ^8^ ^)^, child malnutrition continues to pose a serious public health challenge, with parts of the region now facing a double burden of malnutrition with increasing rates of overweight and obesity as well as persistent undernutrition in under-5s^(^ ^9^ ^)^.

Globally, 3·1 million under-5s die each year because of poor nutrition^(^ ^10^ ^)^. The first 2 years of a child’s life are the most sensitive period to growth faltering^(^ ^11^ ^,^ ^12^ ^)^ and infant and young child feeding practices should adapt to evolving nutritional needs^(^ ^5^ ^)^. Exclusive breast-feeding is recommended for the first 6 months of life and appropriate complementary feeding between the ages of 6 and 23 months^(^ ^12^ ^)^. Further, adequate dietary diversity in early life may influence taste preference and dietary choice in adolescence and early adulthood^(^ ^13^ ^,^ ^14^ ^)^.

Dietary diversity increases the intake of micronutrients and energy in young children^(^ ^15^ ^,^ ^16^ ^)^. Minimum dietary diversity (MDD) is defined as the consumption of at least four out of the following seven food groups: (i) grains, roots, tubers; (ii) legumes and nuts; (iii) dairy products; (iv) meats and fish; (v) eggs; (vi) vitamin-A rich fruits and vegetables; and (vii) other fruits and vegetables^(^ ^17^ ^)^. While 60 % of children aged 6–23 months in the South-East Asia region meet the MDD, the dietary diversity gap between rich and poor is the starkest globally^(^ ^5^ ^)^. Furthermore, dietary diversity is a particular concern due to the traditionally rice-based and vegetarian diets which contribute to micronutrient deficiencies throughout the region, specifically Fe, Zn, vitamin A, iodine and Ca deficiencies^(^ ^18^ ^)^.

The associations between socio-economic status and growth during childhood have been well documented^(^ ^15^ ^,^ ^19^ ^,^ ^20^ ^)^, with children from the richest households and usually those residing in urban areas typically exhibiting better growth; little is known about intra-urban and intra-rural socio-economic disparities. It is predicted that, by 2050, 64 % of the region’s population will live in urban areas^(^ ^21^ ^)^, which has the potential to exacerbate intra-urban socio-economic differentials in child nutritional status. In addition, with over two-thirds of females active in employment in this region, investigating the relationship between maternal employment and MDD in countries experiencing rapid urban transformation is a current and pressing issue^(^ ^22^ ^)^.

In the present paper we examine the socio-economic differentials in MDD among children aged 6–23 months in three economically diverse countries of South-East Asia: Cambodia, Myanmar and Indonesia, which together account for 51 % of the total population of South-East Asia^(^ ^23^ ^)^. Myanmar and Cambodia are low-income countries^(^ ^24^ ^)^, with just over one-third of under-5s being stunted (one of the highest rates among all ASEAN (Association of Southeast Asian Nations) countries^(^ ^9^ ^)^) and under-5 mortality rates that would need to be reduced further to meet the 2030 target^(^ ^25^ ^)^. Indonesia is the most populated lower-middle-income country in the region, with a per capita income approximately three times that of the other two countries^(^ ^23^ ^)^ and confronted with both increasing obesity and persistent high rates of stunting in under-5s^(^ ^26^ ^)^. Indonesia is close to achieving the 2030 target of under-5 mortality with twenty-six deaths per 1000 live births^(^ ^25^ ^)^. All three countries are committed to achieving the UN Sustainable Development Goal (SDG-2) for ending all forms of malnutrition among under-5s by 2030 through improved food security, nutrition and sustainable agricultural production^(^ ^10^ ^)^.

To the best of our knowledge, there are no systematic cross-national or sub-regional analyses of factors associated with dietary diversity among young children in South-East Asia. Analysing data from three economically and culturally diverse countries in this sub-region, we identify the cross-country similarities and differences in factors associated with infant feeding practices and dietary diversity, and contribute to strengthening the evidence base for policy makers at both the national and sub-regional level.

We hypothesised that: (i) socio-economic differences exist in meeting MDD requirements among under-5s; and (ii) the poorest children in rural areas are more vulnerable to MDD deprivation than their urban counterparts.

## Methodology

Data for the present study were drawn from the individual women’s questionnaire from the most recent Demographic and Health Survey (DHS) conducted in Cambodia^(^ ^27^ ^)^ (2014), Myanmar^(^ ^28^ ^)^ (2015–16) and Indonesia^(^ ^29^ ^)^ (2012). All eligible mothers were asked about the types of food given to their youngest child aged <3 years in the day and night prior to survey^(^ ^30^ ^)^, according to food groups as classified by WHO^(^ ^17^ ^)^. Mothers were read a list of different food types and were asked to respond ‘yes’ if the child had received the food item in the previous day or night, even if this food type was combined with other items. In addition, the surveys collected data on the household, socio-economic and demographic characteristics.

The analysis was based on one eligible woman per household reporting on her youngest, singleton child aged 6–23 months, living with the mother at the time of survey. Total sample size ranged from 2127 children in Cambodia to 1339 in Myanmar and 5193 in Indonesia. DHS surveys employed a standard, stratified, two-stage cluster probability sampling to identify households and respondents who are eligible for interview. The eligible respondents, women aged 15–49 years, were selected randomly from the sampled households within each cluster. Further details on DHS sampling design and survey methodology can be found in the relevant DHS reports for each country^(^ ^27^ ^–^ ^29^ ^)^. The women’s response rate in the survey was 98 % in Cambodia, 96 % in Indonesia and 96 % in Myanmar. The item non-response for selected explanatory variables was minimal, and those with missing data at random were removed from the final analysis (<2 % of all cases).

MDD was defined as the proportion of children aged 6–23 months who received foods from four out of seven recommended food groups within the 24 h prior to interview^(^ ^17^ ^)^.

Factors potentially associated with infant dietary diversity were identified based on the literature on complementary feeding in South-East Asia^(^ ^31^ ^–^ ^34^ ^)^ and with reference to the WHO conceptual framework^(^ ^35^ ^)^. We defined socio-economic status at the level of the individual (mothers’ education and level of participation in the labour force), household (wealth quintile) and spatial (urban/rural residence and geographical region). A composite indicator was created for female labour force participation using principal component analysis^(^ ^36^ ^)^, which transformed five individual variables into one indicator, to account for different aspects of employment. These five variables included employment status in the past 12 months, who the mother reported working for, her occupation, type of earnings and whether she was employed all year, seasonally or occasionally. A household wealth index was also calculated separately for urban and rural areas using principal component analysis, to ensure that those from the poorest households in rural areas were effectively captured. In each country, the geographical regions were re-grouped to reduce the number of categories (Table 1).

**Table 1:** Geographical regions of the study context*

Geographical region	Cambodia	Myanmar	Indonesia
Region 1	Phnom Penh	North Myanmar	Sumatra
Region 2	Plain	East Myanmar	Java
Region 3	Tonle Sap	South Myanmar	Lesser Sunda Islands
Region 4	Coast	West Myanmar	Kalimantan
Region 5	Plateau/mountain	Lower Myanmar	Sulawesi
Region 6		Central Myanmar	Maluku Islands
Region 7			New Guinea

* Data from recent Demographic and Health Surveys in Cambodia (2014)^(^ ^27^ ^)^, Myanmar (2015–16)^(^ ^28^ ^)^ and Indonesia (2012)^(^ ^29^ ^)^.

Child characteristics included age in years, sex, birth order, birth interval and birth weight. In addition, we considered morbidity status of the child, defined as any symptoms of acute respiratory infection, diarrhoea or fever in the two weeks preceding the survey. Maternal characteristics included age in years, marital status and number of antenatal visits. Sex of the household head was included as a household characteristic. Maternal media exposure was computed using principal component analysis, which included how often the respondent read newspapers, listened to radio or watched television; the composite index was categorised as frequent, moderate and limited exposure.

We identified two relevant paternal characteristics likely to be associated with children’s MDD requirement. These included father’s highest education level achieved and his type of occupation (agricultural employment, non-agricultural employment and unemployed).

### Statistical analysis

We used the statistical software package Stata version 14.0 for the statistical analyses. Descriptive statistics were calculated accounting for the complex survey design and applying relevant sample weights. The outcome variable was MDD categorized as a binary variable, coded 0 in cases where the child did not receive the MDD in the 24 h prior to interview and 1 in cases where the child did receive the MDD. We initially considered a two-level (individual and primary sampling unit or cluster) random-intercept model to account for the hierarchical nature of the data set. However, there was no difference in the outcome variable at the cluster level due to small sample size, and hence we considered a fixed-effect binary logistic regression for the multivariate analyses.

The variables that were significantly associated with the outcome variable in the bivariate analysis were included in the final multivariable logistic model. The model-building procedure considered a sequential approach to selecting variables, reflecting on evidence from existing studies. During this iterative process, variables that were not significant in the multivariable model were removed and added one by one to measure their effect on the other covariates. The most parsimonious model for all three countries was determined using the Hosmer–Lemeshow test for goodness-of-fit. To maintain comparability between models, the same explanatory variables were used for each country. Collinearity between variables was tested for using variance inflation factors, which measure the strength of pairwise correlations between variables. The final results are presented as adjusted OR (AOR), with 95 % CI.

Interactions based on theoretical assumptions were tested (between urban/rural residence and wealth; and urban/rural residence and mother’s education). However, these were found to be insignificant and were thus excluded from the final adjusted models.

## Results

Of the total 8364 children, approximately half were girls (49·3 % in Cambodia, 48·5 % in Indonesia and 46·2 % in Myanmar). In Myanmar, 21·4 % of children were fourth or higher births, in Cambodia 15·9 % and Indonesia 12·7 %. Just under half of children were reported with at least one symptom of morbidity in Cambodia and Indonesia, and a third of children in Myanmar (Table 2).

**Table 2:** Characteristics of the study sample (unadjusted percentages and 95 % CI) of South-East Asian children (*n* 8364) aged 6–23 months and their families from three economically diverse South-East Asian countries*

	Cambodia (*n* 2127)		Myanmar (*n* 1339)		Indonesia (*n* 5193)	
Characteristics	%	95 % CI	%	95 % CI	%	95 % CI
Child						
Age (months)						
6–11	34·8	32·3, 37·4	32·6	29·6, 35·8	36·1	34·2, 38·1
12–17	31·4	29·0, 34·0	37·2	34·0, 40·4	32·7	30·9, 34·6
18–23	33·8	31·3, 36·4	30·2	27·3, 33·3	31·2	29·4, 33·0
Sex						
Male	50·7	48·0, 53·4	53·9	50·6, 57·1	51·5	49·5, 53·5
Female	49·3	46·6, 52·0	46·2	42·9, 49·4	48·5	46·5, 50·5
Birth order						
First-born	40·0	37·4, 42·7	35·5	32·4, 38·8	38·3	36·4, 40·3
Second to third	44·1	41·5, 46·8	43·1	39·9, 46·3	49·0	47·0, 51·0
Fourth or more	15·9	13·9, 18·0	21·4	18·9, 24·1	12·7	11·6, 13·9
Birth interval (months)						
No previous birth	40·5	37·9, 43·2	36·2	33·1, 39·4	38·7	36·8, 40·7
<24	7·8	6·4, 9·4	7·3	5·8, 9·2	5·5	4·8, 6·4
≥24	51·7	49·0, 54·4	56·5	53·2, 59·8	55·7	53·8, 57·7
Perceived birth weight						
Smaller than average	10·5	9·0, 12·3	12·3	10·3, 14·6	13·0	11·7, 14·4
Average	55·7	53·0, 58·3	60·9	57·6, 64·1	56·5	54·4, 58·4
Larger than average	33·8	31·3, 36·4	26·8	23·9, 29·9	30·5	28·7, 32·4
Morbidity						
No symptoms	56·9	54·2, 59·6	67·3	64·2, 70·3	51·7	49·7, 53·7
At least one symptom	43·1	40·4, 45·8	32·7	29·7, 35·8	48·3	46·3, 50·3
Maternal						
Age (years)						
15–24	34·9	32·4, 37·5	25·2	22·4, 28·2	28·1	26·4, 29·9
25–34	53·0	50·3, 55·7	51·8	48·6, 55·1	52·2	50·3, 54·2
35–49	12·1	10·4, 14·0	23·0	20·4, 25·8	19·6	18·1, 21·3
Highest educational level						
No education	12·6	10·9, 14·5	15·1	12·9, 17·6	1·8	1·5, 2·3
Primary	51·2	48·5, 53·9	44·7	41·5, 48·0	30·0	28·1, 31·8
Secondary/higher	36·2	33·7, 38·8	40·2	37·0, 43·4	68·2	66·3, 70·1
Marital status						
Married/cohabiting	96·0	94·9, 96·9	96·7	95·3, 97·7	97·8	97·1, 98·3
Widowed/divorced/separated	4·0	3·1, 5·1	3·3	2·3, 4·7	2·2	1·7, 2·9
Active labour force participation						
Not working (past 12 months)	30·4	27·9, 33·0	41·3	38·1, 44·6	54·2	52·2, 56·2
Low	36·3	33·8, 38·9	29·4	26·5, 32·5	27·6	25·9, 29·3
High	33·3	30·8, 35·9	29·3	26·4, 32·4	18·2	16·7, 19·9
Exposure to mass media						
Frequent	39·1	36·5, 41·8	34·7	31·6, 37·9	36·0	34·1, 37·9
Moderate	32·0	29·5, 34·6	37·8	34·6, 41·0	31·4	30·0, 33·3
Limited	28·9	26·6, 31·4	27·6	24·8, 30·6	32·7	30·8, 34·5
Antenatal clinic visits						
0–1	6·4	5·2, 7·8	14·2	12·1, 16·7	3·9	3·3, 4·5
2–4	32·1	29·7, 34·7	38·0	34·8, 41·2	12·5	11·3, 13·7
≥5	61·5	58·9, 64·1	47·8	44·5, 51·1	83·7	82·4, 84·9
Paternal						
Highest educational level						
No education	8·8	7·4, 10·4	16·1	13·9, 18·7	1·0	0·8, 1·3
Primary	44·0	41·3, 46·7	39·5	36·3, 42·8	32·0	30·1, 33·9
Secondary/higher	47·2	44·5, 49·9	44·4	41·1, 47·7	67·0	65·1, 68·9
Occupation						
Not-working	0·3	0·0, 0·9	0·0	0·0, 0·0	1·4	1·1, 1·9
Agricultural	47·1	44·5, 49·8	24·8	22·1, 27·7	21·4	19·9, 22·9
Non-agricultural	52·6	49·9, 55·3	75·2	72·3, 77·9	77·2	75·6, 78·7
Household						
Household wealth						
Poorest	23·8	21·7, 26·2	26·6	23·9, 29·6	16·3	15·0, 17·7
Poorer	18·2	16·3, 20·2	22·9	20·3, 25·8	20·8	19·3, 22·5
Middle	19·7	17·6, 22·0	20·1	17·6, 22·9	20·7	19·1, 22·4
Richer	18·2	16·1, 20·5	5·5	13·3, 18·0	22·4	20·8, 24·2
Richest	20·1	17·9, 22·4	14·9	12·7, 17·4	19·7	18·2, 21·4
Sex of household head						
Male	77·5	75·2, 79·7	85·9	83·5, 88·0	91·4	90·2, 92·5
Female	22·5	20·4, 24·8	14·1	12·0, 16·5	8·6	7·5, 9·9
Spatial						
Residence						
Rural	86·0	84·3, 87·5	74·6	71·6, 77·5	50·8	48·8, 52·8
Urban	14·0	12·5, 15·7	25·4	22·6, 28·4	49·2	47·2, 51·2
Geographical region†						
Region 1	8·7	7·3, 10·3	12·8	10·9, 15·0	23·4	22·1, 24·8
Region 2	35·9	33·2, 38·7	15·5	13·1, 18·3	53·4	51·5, 55·3
Region 3	31·5	29·0, 34·0	9·8	8·6, 11·2	5·7	5·2, 6·3
Region 4	6·0	5·1, 7·0	8·3	7·0, 9·8	6·6	6·1, 7·2
Region 5	18·0	16·3, 19·8	34·1	30·9, 37·5	7·9	7·3, 8·5
Region 6	–	–	19·5	17·0, 22·2	1·2	1·1, 1·4
Region 7	–	–	–	–	1·8	1·5, 2·1

* Data from recent Demographic and Health Surveys in Cambodia (2014)^(^ ^27^ ^)^, Myanmar (2015–16)^(^ ^28^ ^)^ and Indonesia (2012)^(^ ^29^ ^)^.

† See Table 1 for geographical regions of the study context.

Cambodia had the highest proportion of children whose mothers were aged between 15 and 24 years (34·9 %), while this accounted for 28·1 % of children in Indonesia and 25·2 % of children in Myanmar. In all three countries, the proportion of mothers not currently in a partnership or marriage was below 5 %. The percentage of mothers who had achieved secondary or higher education was significantly higher in Indonesia (68·2 %) than in Cambodia (36·2 %) and Myanmar (40·2 %). In Cambodia, 33·3 % of mothers reported having high participation in the labour force, 29·3 % in Myanmar and 18·2 % in Indonesia. Only 1·0 % of fathers in Indonesia had no formal education, 8·8 % in Cambodia and 16·1 % in Myanmar; and over three-quarters were reported to be working in agricultural occupations in Myanmar and Indonesia, while in Cambodia this accounted for just over half of fathers (Table 2).

The majority of children in Cambodia (86·0 %) and Myanmar (74·6 %) resided in rural areas, in contrast to Indonesia where approximately equal proportions resided in urban and rural areas (Table 2).

### Dietary diversity

**Fig. 1:**
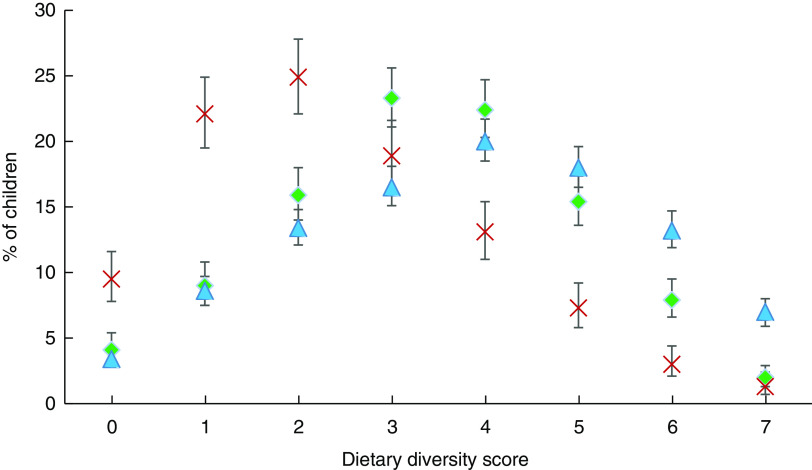
(colour online) Percentage consuming different food groups by country (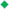, Cambodia; 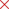, Myanmar; 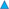, Indonesia), with 95 % CI indicated by vertical bars, among children aged 6–23 months (*n* 8364) from three economically diverse South-East Asian countries. Data from recent Demographic and Health Surveys in Cambodia (2014)^(^ ^27^ ^)^, Myanmar (2015–16)^(^ ^28^ ^)^ and Indonesia (2012)^(^ ^29^ ^)^

Overall, the proportion of children aged 6–23 months reported to be receiving MMD ranged from 24·6 % in Myanmar, to 47·7 % in Cambodia and 58·2 % in Indonesia (Fig. 1).

Foods consisting of grains, roots and tubers featured in the diets of children in all three countries, with over 50 % of children in Cambodia and Myanmar receiving this type of food at age 6 or 7 months respectively, and over 83 % in Indonesia by age 6 months (see online supplementary material, Fig. S1). Flesh foods such as meat and poultry featured in the diets of over half of Cambodian children aged 6–11 months, increasing to 94 % by age 18–23 months. However, only one-third of children aged 6–11 months in Myanmar and Indonesia received flesh foods which increased to 60 % in Myanmar and 70 % in Indonesia by age 18–23 months.

### Factors associated with minimum dietary diversity

Pooling all data from Cambodia, Myanmar and Indonesia (see online supplementary material, Table S1) to quantify the difference between countries in the likelihood of children meeting MDD, with Cambodia as reference category, the odds of meeting MDD was 68 % lower (AOR=0·32; 95 % CI 0·27, 0·38) in Myanmar and 22 % higher in Indonesia (AOR=1·22; 95 % CI 1·08, 1·38). In this pooled analysis, children from the richest households (AOR=2·78; 95 % CI 2·36, 2·92) and those living in urban areas (AOR=1·83; 95 % CI 1·64, 2·04) were more likely to meet the MDD. High labour force participation was associated with a 25 % increased odds of meeting MDD (AOR=1·25; 95 % CI 1·10, 1·42).

### Country-level models

In both Myanmar and Indonesia, children of mothers with secondary education or higher were more likely to receive MDD (AOR=1·39; 95 % CI 1·00, 1·94 and AOR=1·37; 95 % CI 1·18, 1·59, respectively), but this association did not reach statistical significance in Myanmar. Mother’s level of labour force participation was significantly associated with MDD only in Indonesia, with infants of mothers with high participation in the labour force having increased odds of meeting MDD (AOR=1·28; 95 % CI 1·06, 1·53), whereas this association, although in the same direction, did not reach statistical significance in Cambodia and Myanmar (Table 3).

**Table 3:** Factors associated with meeting minimum dietary diversity (adjusted odds ratios (AOR) and 95 % CI) among children (*n* 8364) aged 6–23 months from three economically diverse South-East Asian countries*

	Cambodia (*n* 2096)			Myanmar (*n* 1336)			Indonesia (*n* 5160)		
Characteristics	AOR	95 % CI	*P*	AOR	95 % CI	*P*	AOR	95 % CI	*P*
Socio-economic									
Maternal educational level									
No education/primary	1·00	Ref.	–	1·00	Ref.	–	1·00	Ref.	–
Secondary/higher	1·13	0·90, 1·42	0·277	1·39	1·00, 1·94	0·050	1·37	1·18, 1·59	0·000
Maternal active labour force participation									
Not working (past 12 months)	1·00	Ref.	–	1·00	Ref.	–	1·00	Ref.	–
Low	1·00	0·78, 1·29	0·999	1·11	0·78, 1·59	0·550	1·08	0·93, 1·24	0·324
High	1·10	0·85, 1·41	0·475	1·17	0·82, 1·66	0·382	1·28	1·06, 1·53	0·009
Household wealth									
Poorest	1·00	Ref.	–	1·00	Ref.	–	1·00	v	
Poorer	1·26	0·96, 1·66	0·100	0·99	0·64, 1·53	0·970	1·27	1·05, 1·54	0·014
Middle	1·73	1·28, 2·35	0·000	1·24	0·80, 1·93	0·334	1·53	1·25, 1·87	0·000
Richer	1·16	0·83, 1·62	0·377	0·53	0·96, 2·45	0·075	1·89	1·53, 2·33	0·000
Richest	2·37	1·65, 3·39	0·000	1·81	1·10, 2·96	0·019	1·98	1·58, 2·47	0·000
Residence									
Rural	1·00	Ref.	–	1·00	Ref.	–	1·00	Ref.	–
Urban	1·43	1·10, 1·88	0·008	1·69	1·18, 2·42	0·004	1·66	1·45, 1·90	0·000
Geographical region†									
Region 1	1·00	Ref.	–	1·00	Ref.	–	1·00	Ref.	–
Region 2	0·31	0·18, 0·53	0·000	1·24	0·74, 2·09	0·415	0·97	0·81, 1·16	0·728
Region 3	0·38	0·22, 0·65	0·000	0·72	0·43, 1·21	0·220	0·63	0·49, 0·80	0·000
Region 4	0·33	0·18, 0·60	0·000	0·65	0·37, 1·14	0·131	0·83	0·67, 1·03	0·092
Region 5	0·28	0·16, 0·48	0·000	0·81	0·49, 1·34	0·417	0·55	0·46, 0·67	0·000
Region 6	–	–	–	2·26	1·38, 3·71	0·000	0·55	0·41, 0·73	0·000
Region 7	–	–	–	–	–	–	0·56	0·41, 0·77	0·000
Child									
Age (months)									
6–11	1·00	Ref.	–	1·00	Ref.	–	1·00	Ref.	–
12–17	3·29	2·59, 4·18	0·000	3·28	2·22, 4·84	0·000	3·21	2·77, 3·72	0·000
18–23	3·38	2·60, 4·40	0·000	4·51	2·98, 6·84	0·000	4·74	4·05, 5·56	0·000
Sex									
Male	1·00	Ref.	–	1·00	Ref.	–	1·00	Ref.	–
Female	0·96	0·80, 1·16	0·698	0·70	0·52, 0·93	0·013	1·00	0·88, 1·13	0·999
Breast-feeding status									
Not currently breast-fed	1·00	Ref.	–	1·00	Ref.	–	1·00	Ref.	–
Currently breast-fed	0·73	0·58, 0·92	0·009	0·61	0·42, 0·89	0·010	0·49	0·42, 0·56	0·000
Morbidity									
No symptoms	1·00	Ref.	–	1·00	Ref.	–	1·00	Ref.	–
At least one symptom	0·83	0·69, 1·01	0·065	1·15	0·86, 1·54	0·355	1·25	1·10, 1·42	0·000
Birth interval (months)									
No previous birth	1·00	Ref.	–	1·00	Ref.	–	1·00	Ref.	–
<24	0·60	0·40, 0·89	0·011	0·54	0·30, 1·00	0·048	0·94	0·72, 1·23	0·656
≥24	0·76	0·60, 0·97	0·025	0·70	0·49, 0·98	0·041	0·83	0·71, 0·98	0·032
Maternal									
Age (years)									
35–49	1·00	Ref.	–	1·00	Ref.	–	1·00	Ref.	–
25–34	0·96	0·70, 1·30	0·773	0·57	0·40, 0·82	0·002	0·84	0·71, 1·00	0·044
15–24	0·69	0·48, 0·98	0·039	0·50	0·32, 0·80	0·003	0·73	0·58, 0·90	0·004
Exposure to media									
Frequent	1·00	Ref.	–	1·00	Ref.	–	1·00	Ref.	–
Moderate	0·70	0·56, 0·89	0·004	0·90	0·65, 1·26	0·544	1·47	1·26, 1·72	0·000
Limited	0·60	0·46, 0·79	0·000	0·74	0·49, 1·11	0·147	1·58	1·34, 1·85	0·000

Ref., reference category.

* Data from recent Demographic and Health Surveys in Cambodia (2014)^(^ ^27^ ^)^, Myanmar (2015–16)^(^ ^28^ ^)^ and Indonesia (2012)^(^ ^29^ ^)^.

† See Table 1 for geographical regions of the study context.

Consistently, children from the richest households experienced increased odds of receiving MDD, by approximately twofold or more in each country, while living in an urban area increased the odds (AOR=1·43; 95 % CI 1·10, 1·88 in Cambodia; AOR=1·69; 95 % CI 1·18, 2·42 in Myanmar; AOR=1·66; 95 % CI 1·45, 1·90 in Indonesia; Table 3). Further exploration of the predicted probabilities for children receiving MDD across household wealth quintile, by urban/rural residence (see online supplementary material, Table S2 and Fig. S2), provided insight into intra-urban and intra-rural socio-economic differentials in meeting MDD. Although the differences between the predicted probabilities between children from the poorest and richest households were relatively similar among both urban and rural children, it was clear that the rural poor were consistently disadvantaged, compared with the urban poor.

By geographical region, children residing in households outside the capital city of Phnom Penh in Cambodia experienced decreased odds of meeting MDD. In Myanmar, residing in Central Myanmar (in the regions of Magway, Mandalay and Naypyitaw) was associated with approximately twofold increased odds of receiving MDD (AOR=2·26; 95 % CI 1·38, 3·71; Table 3).

In all three countries children aged 18–23 months were over three times more likely to receive the MDD than those aged 6–11 months. In Myanmar, being a girl was associated with a 30 % decreased odds of meeting MDD (AOR=0·70; 95 % CI 0·52, 0·93), but this association was minimal and not statistically significant in Cambodia and Indonesia (Table 3).

In all three countries, children who were still being breast-fed at the time of the survey were significantly less likely to receive the MDD, with decreased odds of 27 % in Cambodia, 39 % in Myanmar and 51 % in Indonesia, in models adjusting for age of child. Reported symptoms of recent child morbidity increased the odds of meeting MDD in Indonesia by 25 % (AOR=1·25; 95 % CI 1·10, 1·42), but this association was not significant in Cambodia where the odds were decreased and in Myanmar where the odds were increased. A short preceding birth interval (<24 months) was associated with decreased odds of the child receiving MDD in both Cambodia and Myanmar, by approximately half (Table 3).

Children of mothers in the youngest age category (15–24 years) were most at risk for not meeting MDD in all three countries. The association between a composite measure of maternal exposure to media and MDD differed in Cambodia and Indonesia, with limited exposure significantly reducing the odds of meeting MDD by 40 % in Cambodia, non-significantly reducing the odds by 26 % in Myanmar and significantly increasing the odds by 58 % in Indonesia (Table 3).

## Discussion

The findings of our research confirm that urban areas offer advantage over rural areas for meeting MMD in young children^(^ ^37^ ^)^, although intra-urban and intra-rural socio-economic differentials remained.

The finding that maternal higher education was positively associated with MDD in Myanmar and Indonesia is supported by previous research in the Asia-Pacific region^(^ ^31^ ^,^ ^38^ ^–^ ^41^ ^)^. Although maternal education is often considered a proxy for socio-economic status, Ruel *et al*.^(^ ^42^ ^)^ argue that the effect of maternal education on child health and nutrition is independent of socio-economic status, perhaps strengthening the guidance to improve maternal knowledge of optimal child nutrition. Although mother’s labour force participation reached significance only in Indonesia, in all countries children of mothers with a high labour force involvement had an increased likelihood of meeting MDD. Rates of female labour force participation vary across the 6000 islands that constitute Indonesia, but have on the whole remained relatively high compared with other countries in South-East Asia^(^ ^43^ ^)^. Moreover, even among women participating in the labour force, only a very small proportion of women are formally employed in wage jobs^(^ ^43^ ^)^. Our findings highlight that children born to mothers actively engaged in the labour force in Indonesia, i.e. high-status employment in professional or skilled jobs, with job security, year-round employment and wages, were more likely to receive MDD. Unlike previous studies which considered the employment status (whether or not the mother was employed), our study used a composite indicator to understand the effect of maternal labour force participation on dietary diversity in children. This clearly suggests the importance of considering multiple dimensions of female participation in the labour force. This is especially pertinent in countries where many women participate in informal or seasonal employment.

At the household level, in a model allowing for urban/rural setting, there was consistent inequality in the odds of meeting MDD by household wealth quintile in all three countries, with children from the poorest households most at risk of not receiving the MDD, as also shown elsewhere in low- and middle-income countries^(^ ^38^ ^–^ ^41^ ^)^. We also showed a clear link between living in an urban area and improved odds for meeting MDD in children in a model controlling for maternal education and household wealth. The role of urbanisation is important in South-East Asia, a region where 47 % of the population was living in urban areas in 2014, expected to rise to 64 % by 2050^(^ ^21^ ^)^. Urbanisation and the concurrent growth in incomes, employment opportunities and lower food prices perhaps provide advantage over rural areas, where there is a higher dependence on sometimes unpredictable natural resources to meet nutritional needs and lack of national integrated systems for food distribution^(^ ^37^ ^)^.

Finally, we also found that girls were disproportionately at risk of not meeting MDD in Myanmar, despite previous research suggesting no gender differences in infant and young child feeding practices in this country^(^ ^44^ ^)^. Our findings also highlight the need to focus on increasing knowledge on infant and young child feeding practices among younger mothers^(^ ^45^ ^)^. This is particularly pertinent in the South-East Asia region where adolescent birth rates remain high^(^ ^46^ ^)^ and 17·4 % of the population is made up of those aged between 15 and 24 years^(^ ^47^ ^)^. Regular media exposure had a positive effect on meeting MDD in Cambodia, perhaps reflective of the successfulness of the COMBI national campaign 2011–2013 to improve complementary feeding^(^ ^48^ ^)^.

There are some methodological limitations of our study to consider. Limited sample sizes and consequent lack of disaggregated statistics prevented stratified modelling by breast-feeding status; as breast milk is not included in the itemised food groups, it is thus likely that MDD was underestimated among the subgroup of children who were still breast-fed^(^ ^17^ ^)^. Although the use of current-status data may result in overestimating the proportion of children meeting MDD^(^ ^49^ ^)^, use of such data is considered to strengthen the reliability of survey responses due to the reduced recall bias. Finally, high response rates in each country (over 96 % among contacted women) demonstrate the value of DHS data for population-level analysis.

## Conclusion

We confirm the role of urban/rural setting in complementary feeding practices of young children, and further show that socio-economic characteristics of households, mothers and children within both urban and rural areas are influential factors in meeting MDD. Using nationally representative data from three countries in South-East Asia, we have shown that the poorest households in both rural and urban areas are consistently the most disadvantaged and this result is consistent across the sub-region. Using a stratified wealth index that was calculated separately for urban and rural areas, we have tried to ensure that those from the poorest households in rural areas were effectively represented in the current study.

Regardless of location, children of mothers with higher education, better working conditions and higher economic status were more likely to receive MDD. As a result, policies to promote dietary diversity in young children should not only focus on geographical differences, but also target population subgroups from economically disadvantaged communities. Today’s children will become adults by the end of 2030. Therefore, investing in child nutrition and thus development is crucial for achieving Goals 2 and 3 of the 2030 Agenda for Sustainable Development^(^ ^5^ ^)^.

## Supplementary material

For supplementary material accompanying this paper visit https://doi.org/10.1017/S1368980018002173. click here to view supplementary material
