# Supplementary material for: Socio-economic differentials in minimum dietary diversity among young children in South-East Asia: evidence from Demographic and Health Surveys
Source: Public Health Nutr. 2018 Sep 4;21(16):3048–57. doi: 10.1017/S1368980018002173 (PMC6190069; doi:10.1017/S1368980018002173)
Supplement: Supplementary file 1 [file S1368980018002173sup001.docx]

**Supplementary material**

Fig. S1 **Percentage of children aged 6-23 months by food group consumption**

**Cambodia, 2014**

**Myanmar, 2015-16**

Fig. S1 (contd). **Percentage of children aged 6-23 months by food group consumption**

**Indonesia, 2012**

Fig. S2 **Probability of meeting MDD in children aged 6-23 months by household wealth and type of residence**

**Cambodia, 2014**

**Myanmar, 2015-16**

Fig. S2 (contd.) **Probability of meeting MDD in children aged 6-23 months by household wealth and type of residence**

**Indonesia, 2012**

Table S1 **Adjusted odds ratios (95% CI) of factors associated with meeting minimum dietary diversity (pooled analysis)**

| **Characteristics** | **Pooled sample (n=8592)** | | |
| --- | --- | --- | --- |
|  | **AOR** | **95% CI** | ***P*** |
| **Country** |  |  |  |
| Cambodia | 1.00 |  |  |
| Myanmar | 0.32 | (0.27, 0.38) | 0.000 |
| Indonesia | 1.22 | (1.08, 1.38) | 0.000 |
| **Socioeconomic** |  |  |  |
| Highest educational level |  |  |  |
| No education / primary | 1.00 |  |  |
| Secondary / higher | 1.34 | (1.20, 1.50) | 0.000 |
| Labour force participation |  |  |  |
| Not working (past 12 months) | 1.00 |  |  |
| Low | 1.07 | (0.96, 1.20) | 0.238 |
| High | 1.25 | (1.10, 1.42) | 0.001 |
| Household wealth |  |  |  |
| Poorest | 1.00 |  |  |
| Poorer | 1.41 | (1.22, 1.62) | 0.000 |
| Middle | 1.87 | (1.61, 2.17) | 0.000 |
| Richer | 2.09 | (1.79, 2.44) | 0.000 |
| Richest | 2.78 | (2.36, 2.92) | 0.000 |
| Residence |  |  |  |
| Rural | 1.00 |  |  |
| Urban | 1.83 | (1.64, 2.04) | 0.000 |
| **Child** |  |  |  |
| Age (months) |  |  |  |
| 6-11 | 1.00 |  |  |
| 12-17 | 3.17 | (2.82, 3.56) | 0.000 |
| 18-23 | 4.08 | (3.61, 4.62) | 0.000 |
| Sex |  |  |  |
| Male | 1.00 |  |  |
| Female | 0.94 | (0.85, 1.03) | 0.176 |
| Breastfeeding status |  |  |  |
| Not currently breastfed | 1.00 |  |  |
| Currently breastfed | 0.58 | (0.82, 0.65) | 0.000 |
| Morbidity |  |  |  |
| No symptoms | 1.00 |  |  |
| At least one symptom | 1.14 | (1.04, 1.26) | 0.007 |
|  |  |  |  |
|  |  |  |  |

Table S1 (contd.) **Adjusted odds ratios (95% CI) of factors associated with meeting minimum dietary diversity (pooled analysis)**

| **Characteristics** | **Pooled sample (n=8592)** | | |
| --- | --- | --- | --- |
|  | **AOR** | **95% CI** | ***P*** |
| Birth interval (months) |  |  |  |
| No previous birth | 1.00 |  |  |
| <24 | 0.73 | (0.60, 0.89) | 0.002 |
| ≥24 | 0.77 | (0.68, 0.87) | 0.000 |
| **Maternal** |  |  |  |
| Age (years) |  |  |  |
| 35-49 | 1.00 |  |  |
| 25-34 | 0.84 | (0.74, 0.96) | 0.012 |
| 15-24 | 0.67 | (0.57, 0.80) | 0.000 |
| Exposure to media |  |  |  |
| Frequent | 1.00 |  |  |
| Moderate | 1.15 | (1.03, 1.29) | 0.017 |
| Limited | 1.15 | (1.02, 1.29) | 0.022 |
| **Household** |  |  |  |
| Household wealth |  |  |  |
| Poorest | 1.00 |  |  |
| Poorer | 1.41 | (1.22, 1.62) | 0.000 |
| Middle | 1.87 | (1.61, 2.17) | 0.000 |
| Richer | 2.09 | (1.79, 2.44) | 0.000 |
| Richest | 2.78 | (2.36, 2.29) | 0.000 |
| Residence |  |  |  |
| Rural | 1.00 |  |  |
| Urban | 1.83 | (1.64, 2.04) | 0.000 |
| Country |  |  |  |
| Cambodia | 1.00 |  |  |
| Myanmar | 0.32 | (0.27, 0.38) | 0.000 |
| Indonesia | 1.22 | (1.08, 1.38) | 0.002 |

^AOR: Adjusted Odds Ratios; CI: Confidence Interval^

Table S2 **Adjusted predicted probabilities (95% confidence intervals) for children meeting MDD by household wealth and residence**

|  | **Cambodia, 2014** | |  | **Myanmar, 2015-16** | |  | **Indonesia, 2012** | |
| --- | --- | --- | --- | --- | --- | --- | --- | --- |
| **Variable** | ***(N=2096)*** | |  | ***(N=1336)*** | |  | ***(N=5160)*** | |
| Rural |  |  |  |  |  |  |  |  |
| Poorest | 0.36 | (0.31, 0.40) |  | 0.17 | (0.13, 0.21) |  | 0.40 | (0.37, 0.43) |
| Poorer | 0.40 | (0.36, 0.45) |  | 0.17 | (0.13, 0.21) |  | 0.45 | (0.42, 0.48) |
| Middle | 0.47 | (0.42, 0.52) |  | 0.20 | (0.16, 0.24) |  | 0.49 | (0.46, 0.52) |
| Richer | 0.39 | (0.33, 0.44) |  | 0.23 | (0.18, 0.28) |  | 0.54 | (0.50, 0.57) |
| Richest | 0.54 | (0.48, 0.60) |  | 0.26 | (0.20, 0.32) |  | 0.55 | (0.51, 0.58) |
| Urban |  |  |  |  |  |  |  |  |
| Poorest | 0.54 | (0.49, 0.59) |  | 0.30 | (0.23, 0.37) |  | 0.57 | (0.54, 0.61) |
| Poorer | 0.59 | (0.54, 0.64) |  | 0.30 | (0.23, 0.37) |  | 0.62 | (0.59, 0.65) |
| Middle | 0.65 | (0.60, 0.71) |  | 0.34 | (0.27, 0.41) |  | 0.66 | (0.63, 0.69) |
| Richer | 0.57 | (0.51, 0.63) |  | 0.38 | (0.30, 0.46) |  | 0.70 | (0.67, 0.72) |
| Richest | 0.71 | (0.65, 0.77) |  | 0.41 | (0.33, 0.50) |  | 0.70 | (0.67, 0.74) |
